# Expansion of Bragg Reflection Width and Tuning Wavelength in Elastomer-Immobilized Non-Close-Packed Colloidal Crystal Films

**DOI:** 10.3390/polym18080946

**Published:** 2026-04-12

**Authors:** Miyu Makino, Toshimitsu Kanai

**Affiliations:** Graduate School of Engineering Science, Yokohama National University, 79-5 Tokiwadai, Hodogaya-ku, Yokohama 240-8501, Kanagawa, Japan

**Keywords:** colloidal crystals, photonic crystals, elastomers, Bragg reflection width, Bragg reflection wavelength

## Abstract

Colloidal crystals are periodic arrays of monodisperse particles that exhibit optical stopbands, which can be experimentally observed as a Bragg reflection characterized by a specific Bragg wavelength and width. Precise control of these characteristic parameters is essential for applications in structural color materials, sensors, and tunable photonic crystals. Although the Bragg reflection wavelength can be widely tuned by adjusting the lattice spacing via changes in particle size and concentration, controlling the width over a wide range—such as through expansion—is challenging because it is intrinsically determined by the refractive index contrast between the colloidal particles and their surrounding medium. In this study, the Bragg reflection width of non-close-packed colloidal crystals immobilized in an elastomer film was successfully expanded by adjusting the photoinitiator concentration and ultraviolet light intensity for photopolymerization. Expansion was attributed to the superposition of Bragg reflections at different wavelengths, resulting from spatial variations in the lattice spacings of the non-close-packed colloidal crystals formed during photopolymerization. Owing to the solvent-free and highly flexible nature of the elastomer-immobilized, non-close-packed colloidal crystal film, the Bragg reflection wavelength was readily tuned by mechanical compression while maintaining the expanded Bragg reflection width, thereby advancing the practical applications of structural color materials.

## 1. Introduction

Colloidal crystals are three-dimensional ordered arrays composed of monodisperse submicron-sized particles [1,2,3,4]. These crystals exhibit optical stopbands owing to the periodicity of their refractive index and are promising candidates for applications in advanced optical materials, such as nonfading color materials [5,6], sensors [7,8,9,10], and tunable photonic crystals [11,12], which require precise control of the optical stopband. This stopband is characterized by its wavelength and bandwidth, which are experimentally observed as the Bragg reflection wavelength and width, respectively. The Bragg reflection wavelength can be tuned significantly by altering the lattice spacing, according to Bragg’s equation [13]:

(1) $$\lambda_{hkl}=2n_{c}d_{hkl}sin\theta$$

where *λ_hkl_* is the Bragg reflection wavelength, *n*_c_ is the refractive index of the colloidal crystal, *d_hkl_* is the lattice spacing of the (*hkl*) planes, and *θ* is the glancing angle. In opal-type colloidal crystals, where the particles are in contact under dry conditions, the lattice spacing can be varied by changing the particle size [14,15]. In contrast, charge-stabilized colloidal crystals consist of non-close-packed structures of particles suspended in a solvent, stabilized by electrostatic repulsion between particles [16,17]. The lattice spacing of these crystals can be readily tuned by adjusting the particle volume fraction (*ϕ*_p_) rather than the particle size (*d*):

(2) $$d_{hkl}={\left(\frac{2\pi }{3\phi_{p}}\right)}^{1/3}\times \frac{d}{\sqrt{h^{2}+k^{2}+l^{2}}}$$

Furthermore, immobilizing non-close-packed colloidal crystals with a soft polymer allows the lattice spacing to be significantly tuned on demand by applying external stimuli. For instance, the lattice spacing of stimuli-sensitive, gel-immobilized, non-close-packed colloidal crystals can be significantly altered through gel volume changes in response to variations in temperature [18,19,20], pH [21,22,23], or solvent [24,25,26], thereby enabling the tuning of the Bragg reflection wavelength over a wide range. Colloidal crystals immobilized in an elastic polymer—i.e., an elastomer—represent another type of tunable colloidal crystal. In contrast to gel-immobilized systems, these crystals are solvent-free and highly flexible, allowing facile tuning of the lattice spacing and Bragg reflection wavelength via mechanical stress [12,27,28,29].

Although the Bragg reflection wavelength is highly tunable, achieving a substantial change in the Bragg reflection width—particularly via expansion—is challenging, as it is intrinsically determined by the refractive index contrast between the colloidal particles and their surrounding medium, which is difficult to increase appreciably [30]. Previously, multilayered opal-type colloidal crystals composed of different particle sizes were reported to exhibit partial overlap of Bragg reflection peaks from the individual layers [31]. Additionally, opal-type colloidal crystals embedded in a polymer with a graded refractive index distribution displayed a broad reflection width of 112 nm due to the superposition of the shifted Bragg reflection [32]. Although this does not constitute true stopband broadening, it produces an equivalent effect on the apparent spectral properties, which is useful for practical applications. However, the preparation of multilayered colloidal crystals requires monodisperse particles of varying diameters, and the ability to broaden the width in colloidal crystals with a graded refractive index is limited by the low refractive index of the polymer matrix. Our group previously reported that the Bragg reflection width of non-close-packed colloidal crystals immobilized in hydrogel films could be significantly expanded by adjusting the photopolymerization conditions [33,34]. When the gelling agent in charge-stabilized non-close-packed colloidal crystals was photopolymerized under ultraviolet (UV) light for an insufficient duration [33] or with unequal top and bottom light intensities [34], the resulting hydrogel-immobilized colloidal crystal film exhibited a wide Bragg reflection peak, with a width of ~155 nm. This expansion was attributed to the formation of a microscopically inhomogeneous gel network in the film, reflecting the inhomogeneity of the lattice spacing within the non-close-packed colloidal crystals during the subsequent swelling equilibration process.

In this study, we demonstrate, for the first time, that the Bragg reflection width of elastomer-immobilized, non-close-packed colloidal crystal films can be expanded by adjusting the photopolymerization conditions. For this purpose, hydrogel-immobilized, non-close-packed colloidal crystal films with high optical quality are employed as starting materials. The swelling solvent (water) is replaced with an elastomer precursor solution, and the precursor is cured under various photoinitiator concentrations and UV light intensities to generate elastomer-immobilized, non-close-packed colloidal crystal films with expanded Bragg reflection widths. A mechanism for the observed width expansion is proposed. Furthermore, the feasibility of tuning the Bragg reflection wavelength via mechanical compression is examined.

## 2. Materials and Methods

### 2.1. Preparation of the Hydrogel-Immobilized Non-Close-Packed Colloidal Crystal Film

Hydrogel-immobilized, non-close-packed colloidal crystal films were prepared according to a previous method [35]. To prepare the charge-stabilized colloidal crystals, an aqueous dispersion of monodisperse polystyrene particles with a diameter of 160 nm (5016 B, Thermo Fisher Scientific, Waltham, MA, USA) was mixed with 10 wt.% ion-exchange resin (AG501-X8(D), Bio-Rad, Hercules, CA, USA) and gently stirred for two weeks. The obtained charge-stabilized colloidal crystals were subjected to centrifugation (5702RH, Eppendorf, Hamburg, Germany) and mixed with the gelling agent composed of *N*-methylolacrylamide (NMAM, FUJIFILM Wako Pure Chemical Corp., Tokyo, Japan) and *N*-isopropylacrylamide (NIPAM, FUJIFILM Wako Pure Chemical Corp., Tokyo, Japan) monomers, *N*,*N′*-methylenebisacrylamide (BIS, FUJIFILM Wako Pure Chemical Corp., Tokyo, Japan) crosslinker, 2,2′-azobis [2-methyl-*N*-(2-hydroxyethyl)propionamide] (VA, FUJIFILM Wako Pure Chemical Corp., Tokyo, Japan) photoinitiator, and ultrapure water (Merck KGaA, Milli-Q system, Darmstadt, Germany). The concentrations of the polystyrene particles, NMAM, NIPAM, BIS, and VA in this mixture were 15 vol.%, 480 mM, 320 mM, 40 mM, and 0.35 mM, respectively. Colloidal crystals containing the gelling agent were bubbled with argon gas for 5 min and then subjected to shear flow in a flat capillary cell (channel height: 0.1 mm, width: 9 mm, length: 50 mm) to obtain a single-crystalline structure [36]. Subsequently, the gelling agent was subjected to photopolymerization under UV irradiation from the top and bottom faces of the cell for 90 min (MBRL-CUV7530, MORITEX SCHOTT, Saitama, Japan) to immobilize the non-close-packed colloidal crystals in the hydrogel film.

### 2.2. Preparation of the Elastomer-Immobilized Non-Close-Packed Colloidal Crystal Film

The hydrogel-immobilized, non-close-packed colloidal crystal film was cut into ~3 mm-diameter discs, which were then immersed in 4-hydroxybutyl acrylate (4HBA, Tokyo Chemical Industry Co., Ltd., Tokyo, Japan) containing different photoinitiator (DAROCUR 1173, BASF Japan Ltd., Tokyo, Japan) concentrations (*C*_PI_) for 24 h. Silicone oil (KF-96A-50CS, Shin-Etsu Chemical Co., Ltd., Tokyo, Japan) was used as a release agent. Glass slides were coated with silicone oil, and the circular film containing the 4HBA solution was sandwiched between two slides using a 150 µm-thick cover glass spacer. 4HBA was then cured under UV light irradiation (MBRL-CUV7530, MORITEX SCHOTT, Saitama, Japan) at different light intensities (*I*) for 7 h.

### 2.3. Characterization

The reflection spectra and photographic images of the colloidal crystals at normal incidence were recorded using a fiber spectrometer (Fastevert S-2630, Soma Optics, Tokyo, Japan) and a charge-coupled device camera (XCD-V60CR, Sony, Tokyo, Japan), respectively. An elastomer-immobilized, non-close-packed colloidal crystal film, together with silicone oil (KF-96A-50CS, Shin-Etsu Chemical Co., Ltd., Tokyo, Japan), was placed between the two parallel glass substrates of the mechanical compression apparatus. The distance between the glass substrates was adjusted reversibly with an accuracy of ±0.2 μm using three micrometer heads with an attached spring to achieve uniform compression of the elastomer film [37].

## 3. Results and Discussion

**Figure 1:**
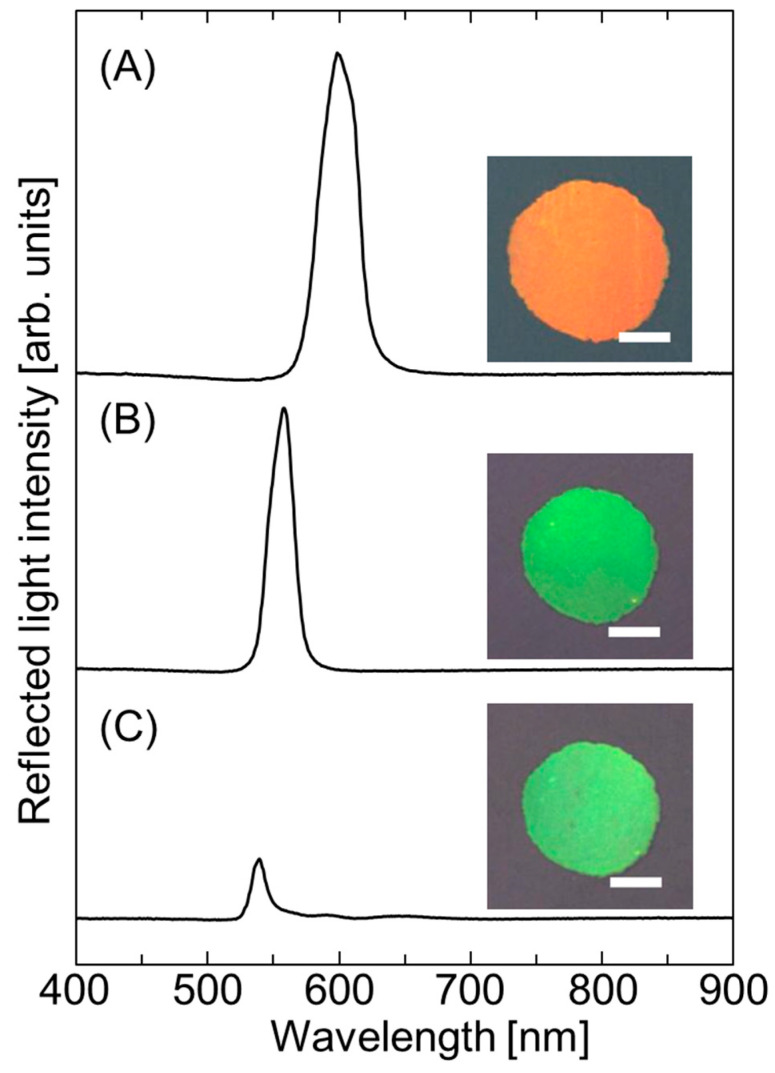
Reflectance spectra and photographic images recorded at normal incidence for the hydrogel-immobilized, non-close-packed colloidal crystal film (**A**) swollen in water, (**B**) after solvent replacement with 4HBA containing 8 wt.% photoinitiator, and (**C**) after UV light irradiation at 100% light intensity. The white bar in the images represents the scale bar of 1 mm.

Figure 1A, 1B and 1C show the reflectance spectra and photographic images recorded at normal incidence for the hydrogel-immobilized, non-close-packed colloidal crystal film swollen with water, after solvent replacement with 4HBA containing 8 wt.% photoinitiator, and after UV light irradiation at 100% light intensity, respectively.

The reflectance spectrum of the water-swollen, hydrogel-immobilized, non-close-packed colloidal crystal film showed a strong peak at 600 nm with a full width at half maximum (FWHM) of 33 nm, corresponding to a uniform orange color (Figure 1A). This peak was attributed to the Bragg reflection from the (111) lattice plane of the face-centered cubic (FCC) structure, which is parallel to the cell surface [36]. Following solvent replacement with 4HBA, the film shrank, producing a green color (Figure 1B). The Bragg reflection peak blue-shifted to 558 nm, and the FWHM reduced to 23 nm. Under UV irradiation, the absorbed 4HBA underwent photopolymerization to yield a solvent-free elastomer-immobilized, non-close-packed colloidal crystal film that exhibited a green color. The Bragg reflection peak blue-shifted to 542 nm after photopolymerization, and the FWHM was 21 nm (Figure 1C). Additionally, the film thickness reduced to 91.2 µm whereas the film diameter remained unchanged because of the glass slides holding the film. The Bragg peak intensity and width of the resultant elastomer-immobilized, non-close-packed colloidal crystal film were smaller than those of the initial hydrogel-immobilized, non-close-packed colloidal crystal film. This was likely due to a reduction in the refractive index contrast [28] and a slight deterioration of the particle arrangement upon photopolymerization.

The peak shift was quantitatively explained by considering the Bragg’s condition. Specifically, the relationship between the Bragg reflection wavelength at normal incidence for the resultant elastomer-immobilized, non-close-packed colloidal crystal film (*λ*^el^) and that for the initial water-swollen, hydrogel-immobilized, non-close-packed colloidal crystal film (*λ*^gel^) can be derived from Equation (3) as follows:

(3) $$\;\;\;\;\lambda^{el}=\frac{n_{c}^{el}}{n_{c}^{gel}}\cdot \frac{d_{hkl}^{el}}{d_{hkl}^{gel}}\lambda^{gel}$$

where $n_{c}^{gel}$ and $n_{c}^{el}$ are the refractive indices of the hydrogel- and elastomer-immobilized, non-close-packed colloidal crystal films, respectively, and $d_{hkl}^{gel}$ and $d_{hkl}^{el}$ are the lattice spacings of the (*hkl*) planes of the corresponding films, respectively. The value of $n_{c}^{gel}$ can be approximated by the volume-weighted average of the refractive indices of its constituent components as follows:

(4) $$n_{c}^{gel}=n_{p}\phi_{p}^{gel}+n_{pol}\phi_{pol}^{gel}+n_{w}\left(1-\phi_{p}^{gel}-\phi_{pol}^{gel}\right)$$

where *n*_p_, *n*_pol_, and *n*_w_ represent the refractive indices of the polystyrene particles, the polymer composed of NIPAM and NMAM, and water, respectively. Additionally, $\phi_{p}^{gel}$ and $\phi_{pol}^{gel}$ are the volume fractions of the polystyrene particles and the polymer composed of NIPAM and NMAM in the hydrogel-immobilized, non-close-packed colloidal crystal film, respectively. The value of *n*_pol_ was approximated based on the volume-weighted average of the refractive indices of poly(NIPAM) and poly(NMAM). The relationship between $\phi_{p}^{gel}$ and $\phi_{pol}^{gel}$ can be obtained from the masses of the particles and gelling agents added to the dispersion. Similarly, $n_{c}^{el}$ is given by:

(5) $$n_{c}^{el}=n_{p}\phi_{p}^{el}+n_{pol}\phi_{pol}^{el}+n_{el}\left(1-\phi_{p}^{el}-\phi_{pol}^{el}\right)$$

where $\phi_{p}^{el}$ and $\phi_{pol}^{el}$ are the volume fractions of the polystyrene particles and the polymer composed of NIPAM and NMAM in the elastomer-immobilized, non-close-packed colloidal crystal film, respectively. The values of $\phi_{p}^{el}$ and $\phi_{pol}^{el}$ were estimated using the $\phi_{p}^{gel}$ value together with the areas and thicknesses of the hydrogel and elastomer films. The value of $d_{hkl}^{el}/d_{hkl}^{gel}$ was estimated as the ratio of the thickness of the elastomer-immobilized, non-close-packed colloidal crystal film to that of the hydrogel-immobilized, non-close-packed colloidal crystal film. By substituting the observed *λ*^gel^ value into Equation (1) and employing Equations (1)–(3), the *λ*^el^ value was obtained as 549 nm, consistent with the observed Bragg wavelength (542 nm).

**Figure 2:**
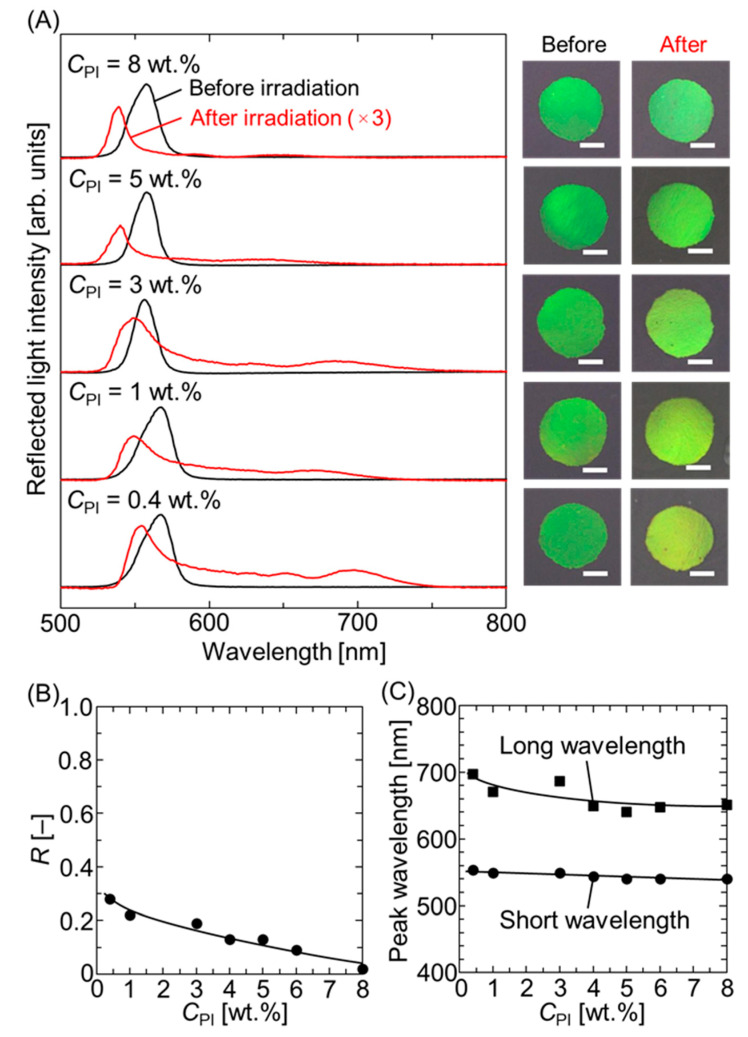
(**A**) Reflectance spectra and photographic images recorded at normal incidence for the hydrogel-immobilized, non-close-packed colloidal crystal films swollen in 4HBA with different *C*_PI_ before and after UV light irradiation at *I* = 100%. The red lines have been magnified three times. The white bar in the images represents the scale bar of 1 mm. (**B**) Ratio of the peak intensity on the long-wavelength side to that on the short-wavelength side, *R*, and (**C**) peak wavelengths on the long- and short-wavelength sides as a function of *C*_PI_.

Figure 2A shows the reflectance spectra and photographic images recorded at normal incidence for the hydrogel-immobilized, non-close-packed colloidal crystal films swollen with 4HBA before and after UV light irradiation at different photoinitiator concentrations (*C*_PI_) and a constant UV light intensity (*I*) of 100%. Upon reducing *C*_PI_ from 8 wt.% to 0.4 wt.%, the spectrum recorded after irradiation showed a gradual increase in the reflected light intensity at longer wavelengths, resulting in a color change in the elastomer-immobilized, non-close-packed colloidal crystal film from green to yellow-green. The ratio of the peak intensity on the long-wavelength side to that on the short-wavelength side (*R*), along with the peak wavelengths on both the long- and short-wavelength sides, is plotted as a function of *C*_PI_ in Figure 2B and 2C, respectively. The *R* value increased from 0.02 to 0.28 when *C*_PI_ was reduced from 8 wt.% to 0.4 wt.%. Both the long- and short-wavelength peak positions increased with decreasing *C*_PI_, and the difference between the peak wavelengths became larger. These spectral red shifts suggest an increase in the lattice spacing of the non-close-packed colloidal crystals.

**Figure 3:**
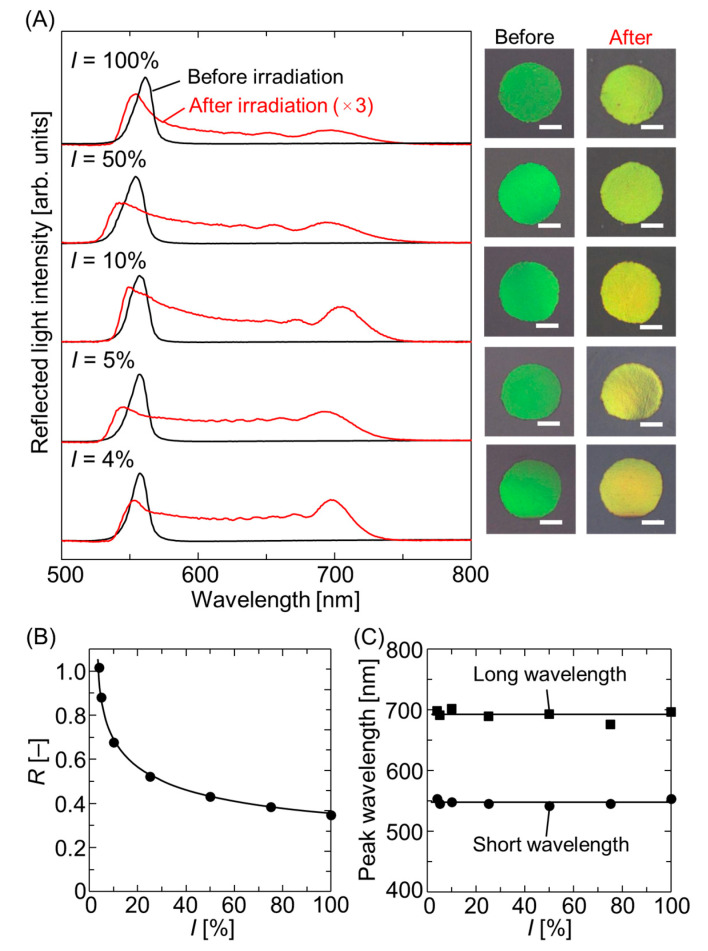
(**A**) Reflectance spectra and photographic images recorded at normal incidence for the hydrogel-immobilized, non-close-packed colloidal crystal films swollen in 4HBA with a constant *C*_PI_ of 0.4 wt.% before and after UV light irradiation at different values of *I*. The red lines have been magnified three times. The white bar in the images represents the scale bar of 1 mm. (**B**) Ratio of the peak intensity on the long-wavelength side to that on the short-wavelength side, *R*, and (**C**) peak wavelengths on the long- and short-wavelength sides as a function of *I*.

To further increase the width and reach an *R* value of 1, *I* was reduced during photopolymerization at a constant *C*_PI_ of 0.4 wt.%. As shown in Figure 3A, upon decreasing *I* from 100% to 50%, 10%, 5%, and 4%, the reflected light intensity on the longer wavelength side increased, and the color changed from yellow-green to yellow. At *I* = 4%, the peak intensity on the long-wavelength side eventually exceeded that on the short-wavelength side, where the *R* value reached 1.01 (Figure 3B). As presented in Figure 3C, the peak wavelengths on both the short- and long-wavelength sides remained nearly constant despite changes in light intensity, indicating the limit of Bragg reflection width expansion. However, the FWHM was determined to be 179 nm at *I* = 5%, reflecting the maximum value reported to date.

**Figure 4:**
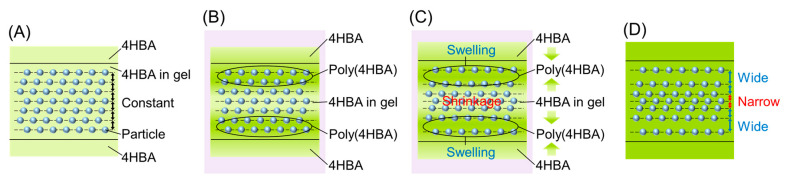
Schematic representation of a probable mechanism for the generation of spatial variation in the lattice spacing. (**A**) Before irradiation, (**B**) during irradiation (coexistence of poly(4HBA) and unreacted 4HBA), (**C**) during irradiation (swelling–shrinking process), and (**D**) after irradiation.

Expansion of the Bragg reflection peak width can be accounted for considering the following mechanism. Firstly, before UV irradiation, the colloidal particles are uniformly spaced while being immobilized in the hydrogel network swollen with the 4HBA precursor solution (Figure 4A).

At a high photoinitiator concentration and high UV light intensity, radicals are rapidly generated at numerous sites, leading to the rapid development of a 4HBA polymer network. Consequently, the particle arrangement is immobilized by poly(4HBA) while maintaining uniform lattice spacing, accompanied by a slight contraction. However, at low photoinitiator concentrations and low UV light intensities, radical generation is slow, and the number of initiation sites is small. Consequently, the 4HBA polymer network develops gradually. During this process, regions of poly(4HBA) and the unreacted 4HBA precursor solution coexist (Figure 4B). Consequently, poly(4HBA) absorbs the surrounding precursor solution, leading to swelling, which results in a wide lattice spacing of non-close-packed colloidal crystals (Figure 4C). In contrast, the lattice spacing of the non-close-packed colloidal crystals in the region of the unreacted 4HBA precursor solution becomes narrower due to absorption of the precursor solution by poly(4HBA). This generates spatial variations in the lattice spacing, which are immobilized in the poly(4HBA) film after sufficient irradiation time (Figure 4D). These spatial variations are manifested as an expansion of the Bragg reflection width toward both the short- and long-wavelength sides, resulting from the superposition of Bragg reflections at multiple wavelengths.

**Figure 5:**
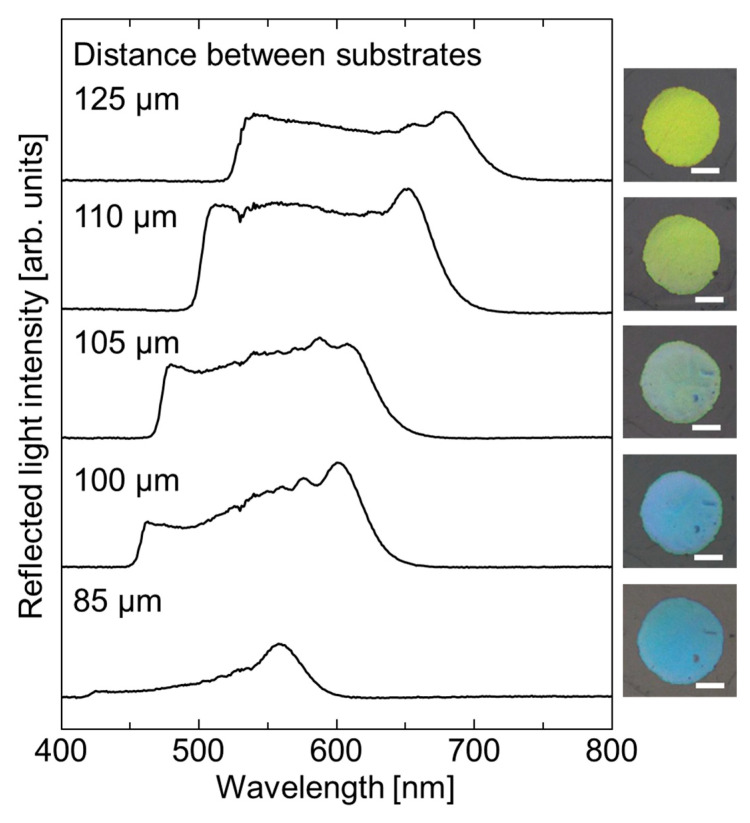
Reflectance spectra and photographic images recorded at normal incidence for the elastomer-immobilized, non-close-packed colloidal crystal film with expanded Bragg reflection width at different substrate separations. The white bar in the images represents the scale bar of 1 mm.

Because of the solvent-free and highly flexible nature of the elastomer-immobilized, non-close-packed colloidal crystal film, the Bragg reflection wavelength can be readily tuned through the application of mechanical compression. Figure 5 shows the reflectance spectra and photographic images recorded for the elastomer films under different degrees of uniaxial compression.

When the distance between substrates was reduced from 125 to 85 µm during compression, the film area and thickness increased and decreased, respectively, and the Bragg reflection wavelength underwent a blue shift of ~150 nm while preserving its width. Additionally, the reflection color changed from yellow to blue. Upon removal of the compressive force, the Bragg wavelength and color returned to their initial values. Such reversible tuning over a wide wavelength range can be attributed to the large interparticle separation of the non-close-packed colloidal crystals immobilized in the elastomer film.

## 4. Conclusions

In this study, the Bragg reflection width of elastomer-immobilized, non-close-packed colloidal crystal films was expanded through careful tuning of the photopolymerization conditions. Upon reducing the photoinitiator concentration and UV light intensity, the spectrum of the resulting elastomer-immobilized, non-close-packed colloidal crystal film confirmed a gradual increase in the reflected light intensity at longer wavelengths. At a photoinitiator concentration of 0.4 wt.% and UV light intensity of 5%, the reflected light intensities on the long- and short-wavelength sides became comparable and the FWHM reached 179 nm, representing the highest value reported to date. The observed expansion was attributed to the superposition of Bragg reflections at multiple wavelengths, resulting from spatial variation in the lattice spacing of the non-close-packed colloidal crystals formed during slow photopolymerization. To verify this hypothesis, it is necessary to directly observe the non-close-packed particle structure immobilized on the elastomer. At this stage, direct observation by using a scanning electron microscope was difficult due to damage to the surrounding soft polymer caused by placing the sample under a vacuum and subjecting it to electron beam irradiation. Confocal microscopy might be effective for observation. Investigation of polarization dependence remains a challenge for future studies. Although we used 3 mm-diameter discs for this study, similar width expansions are to be expected in larger sample sizes. Furthermore, changing the type of polymer potentially affects width expansion and width control. Machine learning approaches [38] may further enhance the study of width control. Owing to the solvent-free and highly flexible nature of the elastomer-immobilized, non-close-packed colloidal crystal film, the Bragg reflection wavelength blue-shifted by ~150 nm under mechanical compression while maintaining the expanded Bragg reflection width. Such width expansion and wavelength tuning are expected to advance practical applications of structural color materials, including multicolor systems, wide-band optical filters, and tunable photonic crystals.

## Data Availability

The original contributions presented in this study are included in the article. Further inquiries can be directed to the corresponding author.

## Abbreviations

The following abbreviations are used in this manuscript:

UV	Ultraviolet
4HBA	4-hydroxybutyl acrylate
NMAM	*N*-methylolacrylamide
NIPAM	*N*-isopropylacrylamide
BIS	*N*,*N′*-methylenebisacrylamide
VA	2-methyl-*N*-(2-hydroxyethyl)propionamide
FCC	Face-centered cubic
